# Antisense oligonucleotide‐mediated knockdown of *Mpzl3* attenuates the negative metabolic effects of diet‐induced obesity in mice

**DOI:** 10.14814/phy2.14853

**Published:** 2021-05-15

**Authors:** Beth L. Worley, Thomas Auen, Amy C. Arnold, Brett P. Monia, Nadine Hempel, Traci A. Czyzyk

**Affiliations:** ^1^ Department of Anesthesiology & Perioperative Medicine Penn State University College of Medicine Hershey PA USA; ^2^ Department of Pharmacology Penn State University College of Medicine Hershey PA USA; ^3^ Biomedical Sciences Program Penn State University College of Medicine Hershey PA USA; ^4^ Department of Neural & Behavioral Sciences Penn State University College of Medicine Hershey PA USA; ^5^ Ionis Pharmaceuticals Inc. Carlsbad CA USA

**Keywords:** beta‐oxidation, cholesterol, diet‐induced obesity, ferredoxin reductase, insulin resistance, non‐alcoholic fatty liver disease

## Abstract

Previously, we demonstrated that global knockout (KO) of the gene encoding myelin protein zero‐like 3 (*Mpzl3*) results in reduced body weight and adiposity, increased energy expenditure, and reduced hepatic lipid synthesis in mice. These mice also exhibit cyclic and progressive alopecia which may contribute to the observed hypermetabolic phenotype. The goal of the current study was to determine if acute and peripherally restricted knockdown of *Mpzl3* could ameliorate the negative metabolic effects of exposure to a high‐fat and sucrose, energy‐dense (HED) diet similar to what was observed in global *Mpzl3* KO mice in the absence of a skin phenotype. *Mpzl3* antisense oligonucleotide (ASO) administration dose‐dependently decreased fat mass and circulating lipids in HED‐fed C57BL/6N mice. These changes were accompanied by a decrease in respiratory exchange ratio, a reduction in energy expenditure and food intake, a decrease in expression of genes regulating de novo lipogenesis in white adipose tissue, and an upregulation of genes associated with steroid hormone biosynthesis in liver, thermogenesis in brown adipose tissue and fatty acid transport in skeletal muscle. These data demonstrate that resistance to the negative metabolic effects of HED is a direct effect of *Mpzl3* knockdown, rather than compensatory changes that could be associated with deletion of *Mpzl3* during development in global KO mice. Inhibiting MPZL3 could be a potential therapeutic approach for the treatment of obesity and associated dyslipidemia.

## Graphical abstract

This study investigated whether acute antisense oligonucleotide (ASO)‐mediated knockdown of Mpzl3 couldrecapitulate the lean and hypermetabolic phenotypes observed in mice withglobal Mpzl3 KO. Mpzl3 ASOprevented the body weight gain, increase in adiposity, glucose intolerance andhyperlipidemia typically associated with exposure to a high‐fat and sucrose, energy‐dense diet. Metabolic improvements were accompanied by anincrease in whole‐body fat oxidation, a reduction in serum lipids andtissue‐specific changes in expression of genes regulating lipogenesis, adaptive thermogenesis, steroid biosynthesis and fatty acid oxidation.

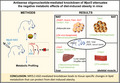

**New and newsworthy**

Antisense oligonucleotide‐mediated peripherally restricted knockdown of myelin protein zero‐like 3 (*Mpzl3*) in mice increased whole‐body fat oxidation and resulted in tissue‐specific changes in genes regulating lipid metabolism in adipose tissue, skeletal muscle and liver. Cumulatively, these changes conferred protection against increased adiposity and hyperlipidemia associated with diet‐induced obesity. The ability of *Mpzl3* deletion to ameliorate the negative metabolic effects of a western diet is not due to the overt fur loss and skin abnormalities previously observed in global knockout mice.

## 1 INTRODUCTION

Obesity is a global health epidemic in developed countries such as the United States, with over 36% of adults and 17% of children and adolescents considered obese (Ogden et al., 2016). Despite initiatives to increase awareness of the detrimental health consequences associated with obesity, these numbers continue to rise with prevalence expected to reach 50% of the adult US population by the year 2030 (Wang et al., 2011). Western diets rich in fat and refined carbohydrates are a major contributor to obesity, leading to multiple comorbid conditions including type 2 diabetes and insulin resistance (Kahn et al., 2006), cardiovascular disease, dyslipidemia, and hypertension, many of which can benefit from weight loss (Hill, 2006). The physiological response to loss of excess body weight (BW) includes improved whole‐body metabolic efficiency (Dulloo & Girardier, 1993; MacLean et al., 2004), decreased energy expenditure (Leibel et al., 1995; Ravussin et al., 1985), and increased food intake (Cornier et al., 2004; Doucet et al., 2000), all of which eventually work against the long‐term maintenance of BW. Therefore, many individuals experience weight regain within a few years if healthful diet and exercise are not maintained (Anderson et al., 2001; Saris, 2001). In addition, recent anti‐obesity drugs have largely failed due to limited efficacy and adverse cardiovascular and other off‐target effects (Srivastava & Apovian, 2018). Currently, the cellular mechanisms underlying physiological changes to weight loss are poorly understood, hindering the development of safe and effective pharmacological therapies. Thus, there is an unmet need to identify new pathways regulating energy homeostasis.

Myelin protein zero‐like 3 (MPZL3) is a novel protein that can regulate energy homeostasis in preclinical models of obesity. The *Mpzl3* gene encodes a single transmembrane protein with an immunoglobulin (Ig)‐like variable (V)‐type domain, and was named for its sequence homology to myelin protein zero (MPZ) and myelin protein zero‐like 2 (MPZL2, also called EVA1; Cao et al., 2007). The presence of an IgV domain suggests that MPZL3 may be involved in cellular process such as cell adhesion, cell‐cell interaction, and antigen binding (Teichmann & Chothia, 2000; Vogel et al., 2003); however, the biological functions of MPZL3 remain largely unknown. We previously demonstrated that MPZL3 plays a critical role in energy balance, as global *Mpzl3* knockout (KO) mice have reduced BW and adiposity which are largely driven by an increase in whole‐body energy expenditure and a pronounced reduction in hepatic lipid synthesis (Czyzyk et al., 2013). Furthermore, *Mpzl3* KO mice had reduced blood glucose levels and an enhanced response to insulin. These collective metabolic changes contribute to resistance of these mice to diet‐induced obesity (Czyzyk et al., 2013). Thus, we identified potential novel physiological roles for MPZL3 in BW regulation, energy expenditure, glycemic control, and hepatic lipid synthesis. Similar reductions in BW and adiposity were subsequently observed in an independently‐generated global *Mpzl3* KO mouse line (Leiva et al., 2014). Interestingly, loss of MPZL3 function has also been shown to cause various skin abnormalities including sebaceous gland hypertrophy and progressive cyclic alopecia (Leiva et al., 2014; Wikramanayake et al., 2016). Fur loss in mice has systemic metabolic consequences, increasing metabolic rate by 50%–300% to counteract heat loss (Hirata et al., 2011; Nedergaard & Cannon, 2014). Furthermore, impaired epidermal lipid metabolism has extensive but less recognized effects, including activation of brown adipose tissue (BAT), increased browning of white adipose tissue (WAT), and increased energy expenditure (Kruse et al., 2017). Thus, increased energy expenditure and overall reduction in lipids in *Mpzl3* KO mice may be compensatory to alopecia, rather than reflective of direct effects of *Mpzl3* loss on systemically regulated metabolic changes.

The goal of the current study was to test whether acute antisense oligonucleotide (ASO)‐mediated knockdown of *Mpzl3* could recapitulate the lean and hypermetabolic phenotypes observed in mice with global *Mpzl3* KO, and similarly lead to amelioration of the negative metabolic effects of exposure to a high‐fat and sucrose, energy‐dense diet (HED). We report that in the absence of alopecia, *Mpzl3* ASO‐mediated knockdown effectively recapitulated several of the phenotypes observed in global *Mpzl3* KO mice fed HED. These findings suggest that *Mpzl3* has effects on metabolism independent of overt effects on skin and other potential compensatory changes from developmental deletion of *Mpzl3*. The phenotypes observed herein were not due to knockdown of this gene in the central nervous system (CNS) as these ASOs do not cross the blood‐brain barrier. Furthermore, while reductions in adiposity and lipogenesis are recapitulated after *Mpzl3* ASO treatment, energy expenditure was conversely decreased, highlighting differences in phenotypes between global *Mpzl3* KO mice and those following temporally and peripherally restricted knockdown. Collectively, these studies illustrate the importance of these complementary approaches in elucidating the function of novel metabolic genes.

## 2 MATERIALS AND METHODS

### 2.1 Animals and treatment

All experiments were performed according to procedures approved by the Institutional Animal Care and Use Committee of the Pennsylvania State University College of Medicine. 4‐ to 5‐week‐old male C57BL/6NHsd mice were obtained from Envigo and group housed in a temperature‐controlled room (22°C) with ad libitum access to water and standard rodent chow (Teklad 2018; 18% calories from fat, 3.1 kcal/g; Harlan Laboratories) on a 12:12‐h light‐dark cycle. After a 1‐week acclimation period, standard chow was exchanged for ad libitum access to HED (Teklad 95217; 39.8% calories from fat, 150 g/kg sucrose, 4.3 kcal/g; Harlan Laboratories) to induce obesity. BW was recorded once per week. After 7‐weeks on HED, these now diet‐induced obese (DIO) mice were assigned to either control or treatment groups (*n* = 8/group) via block randomization by BW and housed individually. Mice were treated with a control ASO (141923; Ionis Pharmaceuticals) or an ASO targeting *Mpzl3* (*Mpzl3*‐ASO; 723571; Ionis Pharmaceuticals) at a dose of 50 mg/kg BW twice per week for 8‐weeks. For dose response studies, a separate cohort of experimentally naïve mice were obtained and acclimated as described above. Immediately after ad libitum exposure to HED, mice were treated with control ASO (75 mg/kg) or *Mpzl3* ASO (25, 50 or 75 mg/kg) twice per week for 7‐weeks. All ASOs were dissolved in 0.9% saline and administered systemically by intraperitoneal injection (Zinker et al., 2002). The chemical composition and mechanism of action of ASOs have been described previously (Pandey et al., 2007; Yu et al., 2005) and is briefly detailed in Table 1, along with the oligonucleotide sequence. Mice were fasted for 4‐h and tissues and serum were collected after euthanization and immediately frozen in liquid nitrogen and stored at −80°C for further analysis.

**TABLE 1:** Sequences of antisense oligonucleotides used

Ionis no.	Sequence	Backbone	2′ Sugar modification	Positions of 2′ sugar modification (*)	MW
Mouse Mpzl3 oligonucleotide chemistry					
723571	TCACTCTCTTCAGATCTGCA	Uniform P = S	2'‐O‐Methoxyethyl	*5‐10‐*5	7147.24
Control oligonucleotide chemistry					
141923	CCTTCCCTGAAGGTTCCTCC	Uniform P = S	2'‐O‐Methoxyethyl	*5‐10‐*5	7152.26

### 2.2 Food intake studies

Food intake was measured following 3‐weeks of ASO treatment. Each individual mouse was placed in a clean cage and given five fresh HED pellets that were weighed at the beginning and end of a 24‐h period. This was repeated for a total of five consecutive days, after which food intake values were averaged for *Mpzl3* ASO and control ASO groups.

### 2.3 Whole body composition and metabolic rate

Whole body composition was measured in conscious lightly restrained mice using the minispec LF90 Body Composition Analyzer (Bruker Optics, Inc.). This minispec analyzer is based on time domain nuclear magnetic resonance spectroscopy and provides amounts of fat, lean tissue and free body fluid. Body composition was measured at four different time points during the study: before HED (‐7 weeks), before ASO treatment start (week 0), and following 3‐ and 6‐weeks of ASO treatment. In dose response studies, body composition was measured after 2‐weeks of ASO treatment. Metabolic rate was measured following 6–7 weeks of ASO treatment using an 8‐cage custom indirect calorimetry system (TSE Systems, Inc.). Each individual cage was equipped with infrared beams to detect ambulation, rearing movement, and fine motor movement along the *x*, *y*, and *z* axes. Inlet and outlet ports into each cage monitored O_2_ and CO_2_ levels, enabling calculation of oxygen consumption (VO_2_, ml/h), carbon dioxide production (VCO_2_, ml/h), and respiratory exchange ratio (RER). RER was calculated as the ratio VCO_2_/VO_2_, while energy expenditure was calculated as caloric expenditure through heat (kcal/h/kg bw). Mice were given 5‐days to acclimate to metabolic cages prior to 48‐h calorimetry measurement (last 24‐h used for analysis). A total of 12 animals were divided into two cohorts of six mice for calorimetry studies.

### 2.4 Serum analyses

Blood samples were obtained at 3‐weeks ASO treatment and at time of sacrifice by submandibular bleed and whole trunk collection, respectively. Serum analyses were performed according to manufacturer's instructions using diagnostic kits for cholesterol and triglycerides (Infinity TR13421 and TR22421; ThermoFisher Scientific), glucose (Autokit 439‐90901; Wako Diagnostics), Leptin (mouse, ELISA kit; Invitrogen) and β‐Hydroxybutyrate (Liquicolor 2440‐058; StanBio Laboratory). Colorimetric assays were measured at 490 nm using the iMark Microplate Absorbance Reader (Bio‐Rad).

### 2.5 Glucose tolerance

Following an overnight (~18‐h) fast with free access to water, an IP glucose tolerance test (IPGTT) was performed (2 g/kg; 50% dextrose solution; Sigma) as described previously (Andrikopoulos et al., 2008; Czyzyk et al., 2013). Blood samples were obtained by tail prick at 0 min and 15‐, 30‐, 90‐, and 120‐min post injection and assessed using standard glucose test strips (Freestyle Lite glucometer; Abbott Diabetes Care Inc.). Blood samples were also collected at each timepoint in heparinized capillary tubes, centrifuged and plasma frozen for later analysis. The total area under the curve was calculated using the formula: blood glucose at [(0 + 15 min)*(15 − 0) + (15 + 30 min)*(30 − 15) + (30 + 90 min)*(90 − 30) + (90 + 120 min)*(120 − 90)]/2 (Potteiger et al., 2002). Insulin levels were determined by mouse ultrasensitive ELISA (Alpco). Homeostatic model assessment of insulin resistance (HOMA‐IR) values were calculated using the formula: (fasting blood glucose [mmol/L] × fasting insulin level [mU/L]]/22.5 as previously described (Andrikopoulos et al., 2008).

### 2.6 Hemodynamic measurement and body temperature

Blood pressure and heart rate were measured non‐invasively in conscious mice after 8‐week ASO treatment using a tail‐cuff system with volume pressure recording sensor technology (CODA Monitor; Kent Scientific). This system has been previously validated to provide accurate blood pressure measurements compared with radiotelemetry methods (Feng et al., 2008; Wang et al., 2017). Mice were acclimated to the system for 2 days prior to measurements, followed by 1 day of data collection, with the average of at least five measurements reported. Body temperature was measured after 8‐week ASO treatment with a rectal probe thermometer (Acorn Temp JKT Thermocouple Thermometer; Oakton Instruments).

### 2.7 RNA preparation and semi‐quantitative RT‐PCR

Total RNA was extracted from frozen tissue samples (100 mg white adipose, 25 mg all others) and homogenized in 1 ml QIAzol Lysis Reagent (Qiagen) using the TissueLyser II (Qiagen). Lysates were purified using the RNeasy Lipid Tissue Mini Kit (Qiagen) with DNase treatment according to the manufacturer's instructions. RNA concentration was measured using a UV‐Vis spectrophotometer (Nanodrop 2000; ThermoFisher Scientific). cDNAs were prepared using the High‐Capacity cDNA Reverse Transcription Kit (Applied Biosystems) according to the manufacturer's instructions. Semi‐quantitative real‐time RT‐PCR (qPCR) was performed as described previously (Czyzyk et al., 2010) using Taqman gene expression assays (Applied Biosystems). Reactions were carried out using a Quantstudio 12k Flex Real‐Time PCR System (ThermoFisher Scientific) and analyzed using QuantStudio 12k Flex software. All individual samples were measured in triplicate. Data were normalized to 18s ribosomal RNA (RN18S) using the delta‐delta Ct method.

### 2.8 Histopathology

Relevant metabolic tissues were dissected, weighed, and placed in 10% formalin and paraffin‐embedded. Microtome slices (5 µm) were stained using hematoxylin and eosin (H&E) and scored in a blinded manner by a trained pathologist.

### 2.9 WAT analysis

Epididymal WAT sections were H&E stained as described above. Microscope images were taken at 20× and the number of cells and relative size (pixel area) were determined using the Adiposoft plug‐in for Fiji NIH Image J2 software (Galarraga et al., 2012; Schindelin et al., 2012). Analysis settings used were as described in Choi et al. (2020).

### 2.10 Data analysis and statistics

Statistical analyses were performed using GraphPad Prism 6 (GraphPad Software). One‐way ANOVA, two‐way ANOVA, or repeated measure (RM) ANOVA with Bonferroni's multiple comparisons posthoc test or unpaired *t*‐tests were used as appropriate. ANCOVA analysis was used to account for effects of body mass on food intake and metabolic variables as appropriate. Values are plotted as the mean ± SEM, and differences between groups considered significant when *p* < 0.05.

## 3 RESULTS

### 3.1 *Mpzl3* ASO treatment reduces BW, adiposity, and circulating lipids

**FIGURE 1:**
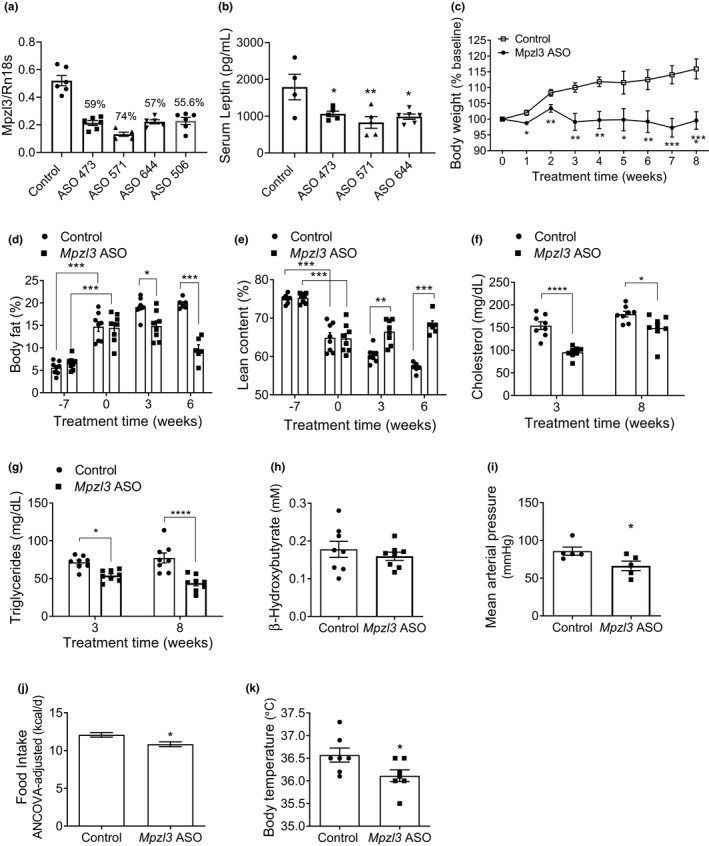
Body weight, adiposity, and circulating lipids are reduced in diet‐induced obese (DIO) mice treated with myelin protein zero‐like 3 (*Mpzl3*) antisense oligonucleotide (ASO). (a) C57BL/6N mice were treated with *Mpzl3* ASOs (50 mg/kg) twice per week for 2‐weeks and semi‐quantitative real‐time RT‐PCR (qPCR) was performed on liver (*n* = 5–6 per group). *Mpzl3* expression was significantly reduced by all ASOs (one‐way ANOVA, *p* < 0.0001). (b) C57BL/6N mice were treated with *Mpzl3* ASOs (50 mg/kg) twice per week for 6‐weeks and serum leptin was measured (*n* = 4–6 per group; one‐way ANOVA, *p* = 0.008). A separate cohort of C57BL/6N DIO mice were treated with control ASO or *Mpzl3* ASO 571 (50 mg/kg) twice per week for 8‐weeks. Body composition was measured using a time domain nuclear magnetic resonance‐based analyzer. (c) Weekly body weights during 8‐weeks ASO treatment period shown as a % of baseline. (d) Body fat as percentage of body weight at 3‐ and 6‐weeks after ASO treatment. (e) Lean mass content as a percentage of body weight at 3‐ and 6‐week ASO treatment. Week‐7 indicates timepoint prior to starting HED and week 0 indicates timepoint just prior to start of ASO injections in both (d) and (e). (f) Serum cholesterol at 3‐ and 8‐week ASO treatment. (g) Serum triglycerides at 3‐ and 8‐week ASO treatment. (h) Serum β‐hydroxybutyrate at 8‐week ASO treatment. (i) Mean arterial blood pressure at 8‐week ASO treatment. (j) Food intake after 3‐week ASO treatment (ANCOVA‐adjusted). Food intake was measured for five consecutive days. Average 24‐h food intake is expressed as kilocalories per day and normalized to body weight. (k) Body temperature after 8‐week ASO treatment as measured by rectal probe. All data expressed as mean ± SEM (*n* = 8/group). Effects of *Mpzl3* ASO treatment and time (c–g) were analyzed by two‐way RM‐ANOVA with Bonferroni posthoc tests. Posthoc: *p < 0.05, **p < 0.01, ***p < 0.001, ****p < 0.0001. Data in (h)–(k) were analyzed with unpaired *t*‐test (*p < 0.05)

To investigate the metabolic effects of acute and peripherally restricted knockdown of *Mpzl3* expression on BW and adiposity, we utilized an ASO approach. Four different *Mpzl3* ASOs (723473, 723506, 723571, 723644) were initially tested for their ability to reduce *Mpzl3* expression in liver compared to a scrambled control ASO (141923). All ASOs reduced *Mpzl3* expression over 50% after 2‐weeks (Figure 1a). In addition, three of these ASOs (723473, 723571, 723644) were tested for their ability to reduce BW. After 6‐weeks, all three ASOs significantly reduced BW (not shown) and serum leptin levels (Figure 1b) in mice fed HED. A non‐treated control group was run in parallel and no differences in BW were observed between this group and the control ASO group after 5‐weeks suggesting that the effects on BW are specific to *Mpzl3* knockdown and not the ASO itself. To more extensively characterize the metabolic effects of *Mpzl3* knockdown, a separate cohort of male C57BL/6N littermates were placed on HED for 7‐weeks to induce obesity (diet‐induced obese, DIO), followed by IP injection of *Mpzl3* ASO (ASO 723571, 50 mg/kg, twice weekly). Within 3‐weeks of treatment, *Mpzl3* ASO prevented the weight gain observed in control ASO treated mice (Figure 1c; two‐way RM‐ANOVA: time effect *p* = 0.007, treatment effect *p* = 0.0021, interaction *p* < 0.0001). Whole body composition analysis demonstrated significant reductions in percent adiposity in *Mpzl3* ASO‐treated animals by 29% within 3‐weeks of treatment (Figure 1d) and 51% by 6‐weeks of treatment compared to controls (Figure 1d; two‐way RM‐ANOVA: time effect: *p* < 0.0001, treatment effect *p* = 0.0004; interaction *p* < 0.0001). Absolute fat mass values were also reduced with *Mpzl3* ASO treatment (two‐way RM‐ANOVA: time effect *p* < 0.0098, treatment effect *p* < 0.0001, interaction *p* = 0.0002; week 0: Control 4.5 ± 0.5 g vs. *Mpzl3* 4.3 ± 0.4 g; 3‐weeks: Control 6.7 ± 0.4 g vs. *Mpzl3* 4.7 ± 0.5 g, posthoc *p* < 0.05; 6‐weeks: Control 7.5 ± 0.3 g vs. *Mpzl3* 3.0 ± 0.4 g, posthoc *p* < 0.01). Relative lean mass as a percentage of BW was elevated in *Mpzl3* ASO‐treated mice compared to controls (Figure 1e; two‐way RM‐ANOVA: time effect *p* < 0.0001, treatment effect *p* = 0.0001, interaction *p* < 0.0001). However, absolute lean mass values of *Mpzl3* ASO‐treated mice were not different when compared to controls (week 0: Control 19.3 ± 0.5 g vs. *Mpzl3* 18.8 ± 0.5 g; 3‐weeks: Control 21.0 ± 0.5 g vs. *Mpzl3* 21.0 ± 0.5 g; 6‐weeks: Control 21.6 ± 0.5 g vs. *Mpzl3* 21.1 ± 0.6 g; two‐way RM‐ANOVA: Time effect, *p* = 0.0002, treatment effect ns *p* = 0.4273, interaction ns *p* = 0.8830). Thus, *Mpzl3* ASO‐treatment significantly reduced HED‐induced gains in adiposity while preserving lean muscle mass throughout the treatment duration.

Given the significant reductions in adiposity in DIO mice treated with *Mpzl3* ASO, we measured serum cholesterol and triglycerides levels at 3‐ and 8‐weeks of treatment. *Mpzl3* ASO reduced serum cholesterol by 38% within 3‐weeks (Figure 1f) and by 17% at 8‐weeks (two‐way RM‐ANOVA: time effect *p* < 0.0001, treatment effect *p* = 0.0002, interaction *p* = 0.0364). *Mpzl3* ASO also reduced serum triglycerides 24% within 3‐weeks (Figure 1g), which was further decreased to 43% at 8‐weeks (two‐way RM‐ANOVA: Time effect ns, *p* = 0.6296, treatment effect *p* = 0.0017). There was no effect of *Mpzl3* ASO on serum β‐hydroxybutyrate (Figure 1h, 8‐weeks).

Previous weight loss agents have been associated with negative cardiovascular effects. In order to determine if *Mpzl3* knockdown adversely impacts cardiovascular function in DIO mice, hemodynamic measurements were obtained after 8‐weeks treatment. Mean arterial pressure was significantly decreased in *Mpzl3* ASO‐treated animals compared to controls, and is consistent with the observed reductions in BW and lipids (Figure 1i). These collective reductions were accompanied by reduced food intake in *Mpzl3* ASO animals (Figure 1j; ANCOVA interaction *p* = 0.0156, *p* < 0.001). A slight but significant decrease in body temperature was also observed in *Mpzl3* ASO animals (Figure 1k; Control 36.57 ± 0.16°C vs. *Mpzl3* ASO 36.11 ± 0.13°C, *p* < 0.05), likely due to pronounced reductions in adiposity. Unlike in the global *Mpzl3* KO mice, we did not see any visible thinning of fur or other evidence of fur loss in this acute knockdown model. Furthermore, analysis of skin biopsies did not reveal any histological differences in *Mpzl3* ASO‐treated mice compared to controls (not shown, *n* = 4). Taken together, these data demonstrate that in the absence of fur loss *Mpzl3* knockdown protects from the negative metabolic effects of HED consumption by promoting a reduction in fat mass, circulating lipids and blood pressure.

### 3.2 *Mpzl3* ASO‐mediated reduction in adiposity and serum lipids is dose‐dependent

**FIGURE 2:**
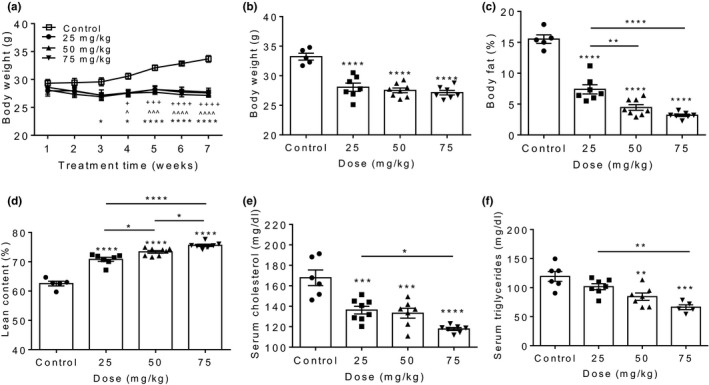
Myelin protein zero‐like 3 (*Mpzl3*) antisense oligonucleotide (ASO) treatment reduced adiposity and circulating lipids in a dose‐dependent manner. Male C57BL/6N mice were placed on a high‐fat and sucrose, energy‐dense diet (HED) and immediately treated with control ASO (50 mg/kg) or *Mpzl3* ASO 571 (25, 50, 75 mg/kg) twice per week for 7‐weeks. (a) Weekly body weights during 7‐week treatment period. (+=25 mg/kg; ^=50 mg/kg; *=75 mg/kg). (b) Average body weights at time of sacrifice. (c) Body fat as percentage of body weight. (d) Lean muscle content as percentage of body weight. Body composition was measured using a time domain nuclear magnetic resonance‐based analyzer after 2 weeks of treatment. (e) Serum cholesterol. (f) Serum triglycerides. Serum was collected at time of sacrifice. Data expressed as mean ± SEM (*n* = 6–8/group). Data in A were analyzed with two‐way RM‐ANOVA and Bonferroni multiple comparisons post‐test; Posthoc *p < 0.05, **p < 0.01, ***p < 0.001, ****p < 0.0001; ^^^ *p* < 0.05, ^^^^^ *p* < 0.001, ^^^^^^ *p* < 0.0001; ^+^ *p* < 0.05, ^+++^ *p* < 0.001, ^++++^ *p* < 0.0001. Data in B‐F were analyzed with one‐way ANOVA and Bonferroni multiple comparisons post‐test; *p < 0.05, **p < 0.01, ***p < 0.001, ****p < 0.0001

To determine whether knockdown of *Mpzl3* expression by ASO exhibits dose‐dependent metabolic effects, we fed a separate cohort of mice HED while simultaneously treating with control ASO (75 mg/kg) or *Mpzl3* ASO (25, 50 or 75 mg/kg) twice per week for 7 weeks (IP). Significant decreases in BW were observed in the 75 mg/kg group after 3 weeks of treatment (Figure 2a; Control 29.58 ± 0.71 vs. *Mpzl3* ASO 26.93 ± 0.47, *p* < 0.05), with all groups exhibiting significant and similar reductions in BW compared to controls after 4 weeks (Figure 2a; two‐way RM‐ANOVA: time effect *p* < 0.0001, treatment effect *p* = 0.0014, interaction *p* < 0.0001). Similar reductions in BW were also observed at all doses at time of sacrifice in *Mpzl3*‐ASO treated mice (Figure 2b; one‐way ANOVA, *p* < 0.0001). Whole body composition measurements conducted after 2 weeks of *Mpzl3* ASO treatment demonstrated dose‐dependent decreases in adiposity, with significant lowering in the 25, 50 and 75 mg/kg groups (Figure 2c, one‐way ANOVA, *p* < 0.0001). We also observed a dose‐dependent increase in relative lean muscle content compared to control mice (Figure 2d; one‐way ANOVA, *p* < 0.0001). As expected, absolute fat mass values were correspondingly reduced after 2‐weeks (Control 5.2 g, 25 mg/kg 2.1 g, *p* < 0.0001; 50 mg/kg 1.3 g, 75 mg/kg 0.9 g; one‐way ANOVA, *p* < 0.0001) and absolute lean mass values were preserved (Control 20.8 g, 25 mg/kg 19.8 g, 50 mg/kg 20.2 g, 75 mg/kg 20.5 g; one‐way ANOVA, *p* = ns).

We next measured circulating lipids and observed significant dose‐dependent reductions in serum cholesterol in all groups compared to controls (Figure 2e; one‐way ANOVA; *p* < 0.0001). Similar decreases in serum triglycerides were found, with significant response in 50 and 75 mg/kg *Mpzl3* ASO‐treated groups compared to controls (Figure 2f; one‐way ANOVA, *p* = 0.0001).

### 3.3 *Mpzl3* ASO improves glycemic control

**FIGURE 3:**
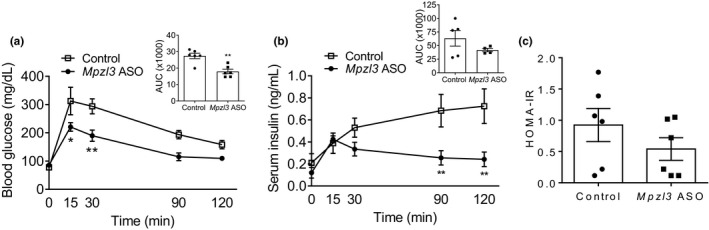
Glycemic control is improved in diet‐induced obese (DIO) mice treated with myelin protein zero‐like 3 (*Mpzl3*) antisense oligonucleotide (ASO). C57BL/6N DIO mice were treated with control ASO or *Mpzl3* ASO (50 mg/kg) twice per week for 8 weeks. At week 7, animals were administered IP 50% dextrose solution at a final concentration of 2 g/kg and subjected to a glucose tolerance test (GTT). (a) GTT after overnight fast. Inset: Area under the curve (AUC) values for GTT. (b) Serum insulin levels at time of GTT. Inset: AUC values for serum insulin. (c) Homeostatic model assessment of insulin resistance values as calculated using the formula (fasting blood glucose [mmol/L] × fasting insulin [mU/L])/22.5. Data expressed as mean ± SEM (*n* = 5–6/group). Data in (a) and (b) were analyzed with two‐way RM‐ANOVA and Bonferroni multiple comparisons post‐test or unpaired *t*‐test; (a) Treatment effect: *p* < 0.01 versus control. Time effect: *p* < 0.0001 versus control. Interaction: ns. (b) Treatment effect: p = 0.0004 versus control. Time effect: p < 0.05 versus control. Interaction: p = 0.0007. Posthoc *p < 0.05, **p < 0.01. Data in C were analyzed with unpaired *t*‐test

Given reductions in adiposity in *Mpzl3* ASO‐treated mice, we measured serum glucose and insulin levels during an IPGTT administered after 7‐weeks of *Mpzl3* ASO treatment in DIO mice. Although fasting blood glucose levels were similar to controls, *Mpzl3* ASO animals exhibited improved glucose tolerance as demonstrated by reduced blood glucose (Figure 3a) levels over time after receiving a bolus glucose injection. Insulin levels at baseline and 15 min post‐glucose injection were not different (Figure 3b) but were reduced in *Mpzl3* ASO treated mice at later time points. Calculated HOMA‐IR values were not significantly different in *Mpzl3* ASO animals (Figure 3c; unpaired *t*‐test, *p* = 0.2592).

### 3.4 *Mpzl3* ASO increases whole‐body fat oxidation and downregulates lipogenesis

**FIGURE 4:**
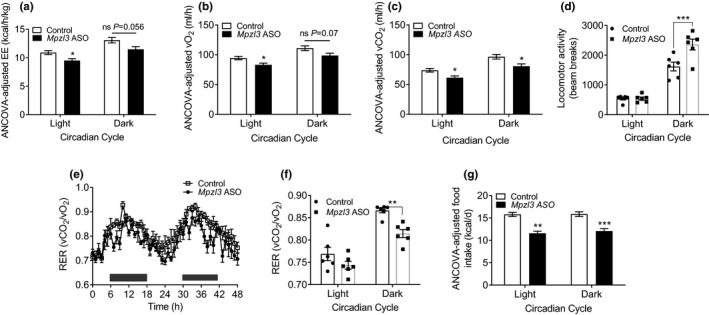
Respiratory exchange ratio is decreased in diet‐induced obese (DIO) mice treated with myelin protein zero‐like 3 (*Mpzl3*) antisense oligonucleotide (ASO). DIO C57BL/6N mice were treated with control ASO or *Mpzl3*‐ASO (50 mg/kg) twice per week for 8 weeks. Indirect calorimetry methods were used after 6–7 weeks treatment. (a) 48‐h energy expenditure. (b) 48‐h VO_2_ levels. (c) 48‐h vCO_2_ levels. (d) 48‐h locomotor activity levels. (e) 48‐h respiratory exchange ratio (RER) levels. (f) Average RER over 48 h. (g) 48‐h food intake. Mice were maintained on a 12:12‐h light‐dark cycle at 23°C. Data expressed as mean ± SEM (*n* = 6/group). Data analyzed with ANCOVA or two‐way ANOVA and Bonferroni multiple comparisons post‐test; Posthoc *p < 0.05, **p < 0.01, ***p < 0.001

We next utilized indirect calorimetry to investigate whether reductions in adiposity were due to altered metabolic rate. *Mpzl3* ASO animals exhibited significantly decreased energy expenditure during the light phase, and trended towards significance in the dark phase (Figure 4a). Similar reductions were observed in vO_2_ levels (Figure 4b) and vCO_2_ levels (Figure 4c) over a 48‐h period. An increase in locomotor activity was observed in *Mpzl3* ASO‐treated animals during the dark phase and thus the reduction in overall energy expenditure was not due to a reduction in activity (Figure 4d; two‐way ANOVA: treatment effect *p* = 0.0067, time effect *p* < 0.0001, interaction *p* = 0.007). Interestingly, we also observed a significant decrease in RER, a measurement of preferred substrate utilization (Figure 4e,f; two‐way ANOVA: treatment effect *p* = 0.0011, time effect *p* < 0.0001, interaction ns *p* = 0.21), which was most pronounced during the dark phase of the circadian cycle. RER values fall on a scale ranging from 0.7 (fat utilization) to 1.00 (carbohydrate utilization). Decreased RER during the dark phase of the circadian cycle, coinciding with increased dark phase locomotor activity, indicates that *Mpzl3* ASO may preferentially induce β‐oxidation, rather than carbohydrate catabolism, during periods of activity. *Mpzl3* ASO animals exhibited a significant reduction in food intake during metabolic studies (Figure 4g). Collectively, an increase in overall fat oxidation, a reduction in food intake and an increase in activity may be expected to contribute to the lean phenotype and overall reduction in circulating lipids in *Mpzl3* ASO treated mice. Moreover, the reduction in overall energy expenditure may be an acute compensatory mechanism in response to reduced food intake and fat mass after *Mpzl3* ASO treatment.

**FIGURE 5:**
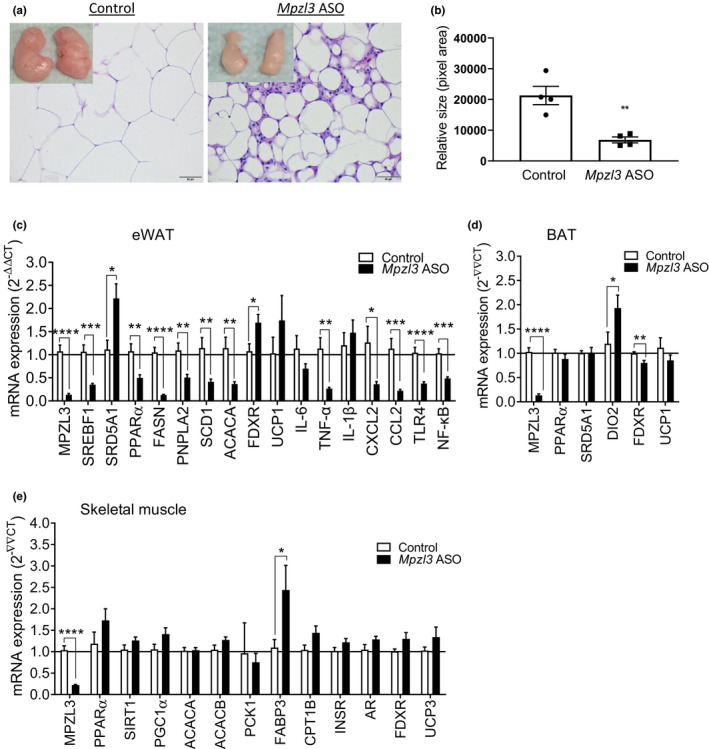
Myelin protein zero‐like 3 (*Mpzl3*) antisense oligonucleotide (ASO) treatment reduces adipocyte size and downregulates genes regulating lipogenesis in white adipose tissue. Male C57BL/6N mice were placed on a high‐fat and sucrose, energy‐dense diet and treated with control ASO or *Mpzl3* ASO (50 mg/kg) twice per week for 8‐weeks. (a) Hematoxylin and eosin‐stained epididymal white adipose tissue (eWAT; 40×). (b) Relative sizes of adipocytes in eWAT were measured with Image J (*p* = 0.004; *n* = 4 per group). Accordingly, the number of adipocytes were significantly increased in *Mpzl3* ASO‐treated mice (not shown; Control ASO: 178 ± 18 cells vs. *Mpzl3* ASO: 406 ± 31 cells, *p* = 0.0007). (c) Semi‐quantitative real‐time RT‐PCR (qPCR) analysis of mRNA expression in eWAT normalized to Rn18s. (d) qPCR analysis of mRNA expression in interscapular brown adipose tissue (BAT). (e) qPCR analysis of mRNA expression in skeletal muscle (quadricep). Data expressed as mean ± SEM (*n* = 4/group). Data analyzed with unpaired *t*‐test; *p < 0.05, **p < 0.01, ***p < 0.001, ****p < 0.0001 compared to control

To investigate the metabolic effects of *Mpzl3* depletion at the cellular level, we conducted histopathological analysis on WAT. Histological analysis of WAT revealed striking reductions in adipocyte size in *Mpzl3* ASO animals compared to controls (Figure 5a,b). In accordance with this finding, there was a significant downregulation in expression of the lipogenic genes *Pparα*, *Fasn*, *Srebf1*, *Pnpla2*, *Scd1*, and *Acaca* in *Mpzl3* ASO mice, while we observed an upregulation in expression of steroid biosynthesis mediators *Srd5a1* and *Fdxr* (Figure 5c). Unexpectedly, we observed a significant increase in what appeared to be crown‐like structures in WAT (Figure 5a) which are typically associated with increased macrophage infiltration and are similar to that found in lipodystrophic mice (Herrero et al., 2010). Consistent with reduced adiposity, expression of several inflammatory cytokine and chemokine genes were either not changed or reduced in *Mpzl3* ASO‐treated mice (Figure 5c). This is in contrast, however, to the increased expression of genes encoding tumor necrosis factor alpha (TNF‐α), interleukin 1 beta (IL‐1β) and interleukin 6 (IL‐6) that were observed in adipose tissue of lipodystrophic mice (Herrero et al., 2010). It can be speculated that these increases in macrophages may be a compensatory mechanism to the rapid catabolism of lipids in these tissues in *Mpzl3* ASO treated mice and may play a role in tissue remodeling. In BAT, we observed a significant increase in expression of *Dio2* (*p* = 0.0007) which is an essential regulator of adaptive thermogenesis in this tissue (Figure 5d). Gene expression analysis of skeletal muscle revealed no differences in *Fdxr*, *Ar*, or *Pparα* mRNA expression levels between groups (Figure 5e). Interestingly, we observed a striking and significant increase in *Fabp3* expression indicating that fatty acid uptake may be stimulated in skeletal muscle of *Mpzl3* ASO animals. Moreover, we also found increases in expression of genes encoding key regulators of fatty acid oxidation and mitochondrial biogenesis CPT1B (*p* = 0.06) and PGC1α (*p* = 0.08), respectively that approached significance in this tissue (Figure 5e). Collectively, these changes are consistent with an increase in fatty acid utilization in skeletal muscle and are in concordance with the observed increase in whole‐body fat oxidation.

**FIGURE 6:**
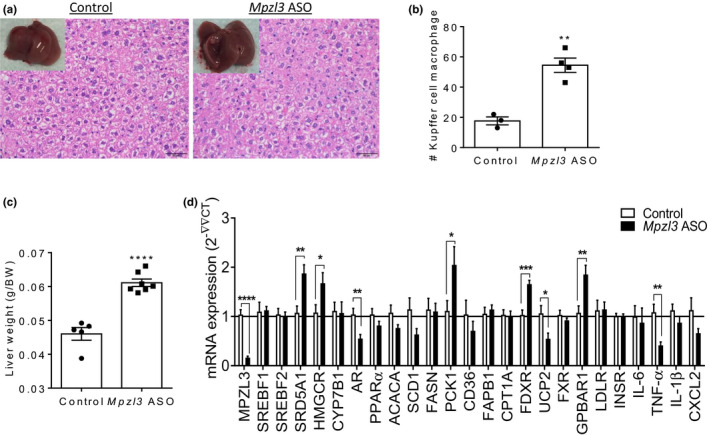
Myelin protein zero‐like 3 (*Mpzl3*) antisense oligonucleotide (ASO) treatment increases histiocytic infiltration of liver tissue and induces changes in genes regulating steroid hormone biosynthesis and bile acid receptor *Gpbar1* in diet‐induced obese mice. Male C57BL/6N mice were placed on a high‐fat and sucrose, energy‐dense diet and treated with control ASO or *Mpzl3* ASO (50 mg/kg) twice per week for 8‐weeks. (a) Hematoxylin and eosin‐stained liver tissue (40×). (b) Kupffer cell hyperplasia was observed in liver tissue of *Mpzl3* ASO‐treated animals. (c) Whole liver weights. (d) Semi‐quantitative real‐time RT‐PCR (qPCR) analysis of mRNA expression in liver normalized to Rn18s. Data expressed as mean ± SEM (*n* = 4/group). Data analyzed with unpaired *t*‐test; *p < 0.05, **p < 0.01, ***p < 0.001, ****p < 0.0001 compared to control

Histological analysis of liver tissue revealed significant Kupffer cell hyperplasia (Figure 6a,b) and increased liver wet weights (Figure 6c) in *Mpzl3* ASO‐treated animals. Gene expression analysis of liver tissue revealed an upregulation in *Fdxr*, *Pck1*, *Hmgcr*, and *Srd5a1* mRNA levels in mice treated with *Mpzl3* ASO (Figure 6d). Since *Hmgcr* (the rate‐limiting enzyme in cholesterol biosynthesis) was upregulated, we measured expression of two regulators of bile acid synthesis and extrahepatic cholesterol catabolism to bile acids, *Fxr* (*Nr1h4*) and *Cyp7b1*. Expression of these genes remained unchanged between groups. However, there was a significant increase in the secondary bile acid receptor *Gpbar1*. The upregulation of cholesterol synthesis, steroid hormone biosynthesis, and bile acid receptor related genes suggests that the observed changes in liver in *Mpzl3* ASO treated mice may be due to a local increase in hepatic cholesterol and its metabolism. Lastly, genes encoding for the pro‐inflammatory cytokines TNFα and IL‐1β were either reduced or not changed, respectively, indicating that the observed increase in Kupffer cells is likely not coupled with an overt increase in inflammation in this tissue **(**Figure 6d).

Conversely, we observed a downregulation in *Ar* and uncoupling protein 2 (*Ucp2*) mRNA levels in livers of *Mpzl3* ASO animals (Figure 6d). *Ar* functions as a steroid‐hormone activated transcription factor (Jänne & Shan, 1991), while an upregulation of *Ucp2* is associated with fatty liver (Rousset et al., 2004). *Scd1* and *Acaca*, involved in fatty acid biosynthesis, had strong downward trends (*p* = 0.07, *p* = 0.08, respectively) in *Mpzl3* ASO animals. *Mpzl3* knockdown of ≥80% by *Mpzl3* ASO was confirmed in all tissues (Figures 5c–e, and 6d). As expected, no changes in *Mpzl3* were observed in hypothalamic brain tissue (not shown) since the ASOs do not penetrate the blood brain barrier (Yu et al., 2005). Overall, gene expression data indicated that there were tissue‐specific changes in lipid metabolism with an overall reduction in de novo lipogenesis, an upregulation in fatty acid oxidation and alterations in cholesterol metabolism in *Mpzl3* ASO treated mice. The above data argues that the physiological changes observed in *Mpzl3* ASO treated mice were driven by cumulative tissue‐specific changes in lipid metabolism resulting in an overall protection from diet‐induced obesity.

## 4 DISCUSSION

The current study has identified ASO‐mediated knockdown of *Mpzl3* (*Mpzl3* ASO) as an approach that validates the potential of targeting MPZL3 therapeutically. *Mpzl3* ASO prevented the BW gain, increase in adiposity, glucose intolerance and hyperlipidemia typically associated with HED intake. We previously showed that *Mpzl3* global KO mice have increased energy expenditure and resistance to the negative effects of HED, including weight gain, hyperlipidemia and hyperglycemia. However, *Mpzl3* KO mice exhibit pronounced fur loss and decreased core body temperature during fasting (Czyzyk et al., 2013), which may contribute to their hypermetabolic phenotype. We previously found that MPZL3 is expressed in both the CNS and in metabolically active tissues including liver, BAT, WAT and skeletal muscle. By acutely and peripherally restricting *Mpzl3* knockdown using ASO, we were able to ameliorate the negative metabolic effects of long‐term exposure to HED, recapitulating many of the global *Mpzl3* KO phenotypes. These effects occurred independently of any overt changes in skin phenotype, but were accompanied by decreases in food intake (Figure 1), indicating a direct consequence of *Mpzl3* knock‐down rather than a compensatory effect due to fur loss. These metabolic improvements were accompanied by a reduction in RER suggesting an increase in whole‐body fat oxidation. Moreover, genes regulating lipogenesis were downregulated with *Mpzl3* ASO treatment in WAT. While increasing mitochondrial fatty acid oxidation alone may not reduce adiposity (Hoehn et al., 2010), the tissue specific effects we observed in *Mpzl3* ASO‐treated mice could have cumulatively led to protection against the weight gain, hyperlipidemia and hyperglycemia normally seen in mice fed HED.

Several limitations to this study should be noted. ASOs themselves may induce metabolic changes as recently described (McCabe et al., 2020) or could cause general toxicity. Perhaps arguing against this, lean body mass was preserved in *Mpzl3* ASO treated mice compared to controls and histopathology was consistent with mice on a high‐fat diet. Conditional KO studies are needed to rule out the possibility that some of our observed phenotypes may be due to potential off‐target effects. In addition, a more thorough investigation of liver function is needed to definitively rule out liver toxicity due to ASO treatment. Furthermore, pair‐feeding and more extensive food intake analysis (i.e., greater than 1 week) would need to be performed to rule‐out that observed phenotypes are not solely being driven by reduced food intake. Lastly, mice were individually housed throughout the 8‐week study period. While single housing allows for individual metabolic parameters to be measured such as food intake and energy expenditure and reduces social hierarchy behaviors among cagemates, it could impact the findings. In general, single housing can be stressful and thermogenesis increased due to the lack of additional warmth that is provided by cagemates. However, the overall impact of single‐housing may be variable and could be influenced by strain (Schipper et al., 2020). In one study, although BWs were lower in single‐housed mice, urinary corticosterone levels were found to be lower after an acclimation period when compared to group‐housed mice (Kamakura et al., 2016). These factors have been mitigated as much as possible during these studies with acclimation time, the inclusion of control ASO mice that were also single‐housed and the use of nestlets in each cage.

We found that *Mpzl3* ASO treatment improved glucose tolerance in HED mice similar to the phenotype of global *Mpzl3* KO mice (Figure 3; Czyzyk et al., 2013). Although whole body insulin sensitivity was not measured directly, *Mpzl3* ASO mice appear to have normal insulin sensitivity as well as intact glucose‐stimulated insulin secretion during IPGTT. Unexpectedly, *Pck1*, a main control point for gluconeogenesis that has been linked to diabetes and obesity (Hanson et al., 1998), was significantly upregulated in livers of *Mpzl3* ASO animals (Figure 6d). The association between poor glycemic control and elevated concentrations of total cholesterol, LDL cholesterol, and triglycerides is well known (Petitti et al., 2007). An overall reduction in lipids, in particular in skeletal muscle, would be expected to improve whole‐body insulin sensitivity and reduce glucose levels. A significant limitation of these measurements was that mice were subjected to an overnight fast and therefore the results do not directly reflect insulin‐mediated glucose uptake (Andrikopoulos et al., 2008; Ayala et al., 2010). Furthermore, it could be argued that *Mpzl3* ASO treated mice received a smaller glucose bolus as they weighed significantly less but had similar skeletal muscle mass to influence the GTT results (Ayala et al., 2010). To further determine the site‐specific effects of *Mpzl3* on whole‐body insulin sensitivity and glucose production a more systematic analysis of glucose homeostasis, including insulin tolerance tests and hyperinsulinemic‐euglycemic clamp studies, will need to be performed in the future (Ayala et al., 2011).

We previously determined that global *Mpzl3* KO mice have food intake‐independent increases in energy expenditure (Czyzyk et al., 2013). Energy expenditure is controlled by several factors, including BAT‐activated thermogenesis, non‐exercise‐induced thermogenesis (i.e., foraging activity), and oxidative capacity of lean skeletal muscle tissue (Czyzyk et al., 2013). MPZL2, the closest MPZL3 homolog, has been shown to be upregulated in BAT compared to less metabolically active tissues such as WAT or beige adipose tissue (Seale, 2015; Wu et al., 2012). Thus, *Mpzl3* and other related genes may play a role in BAT to contribute to overall energy expenditure. However, unlike global *Mpzl3* KO animals, we observed decreased energy expenditure with *Mpzl3* ASO treatment (Figure 4). Interestingly, we also observed decreased RER (Figure 4e,f) indicating that the acute anti‐obesity effects of *Mpzl3* ASO were likely due, in part, to increased whole‐body fat oxidation. It is important to note that we detected *Mpzl3* expression in the hypothalamus of *Mpzl3* ASO animals at levels similar to controls (not shown), and it has been shown previously that ASOs do not efficiently cross the intact blood‐brain barrier (Bennett et al., 2017). Numerous studies indicate CNS regulation of both insulin sensitivity and glucose homeostasis (Xu et al., 2011), food intake, and energy expenditure (Fang et al., 2011). Thus, it is possible that loss of *Mpzl3* in the CNS is driving the increases in energy expenditure observed in global KO mice that were not observed with *Mpzl3* ASO treatment. Furthermore, reductions in energy expenditure and food intake in *Mpzl3* ASO animals may be a compensatory effect resulting from sudden loss of body mass and adiposity. Interestingly, *Mpzl3* ASO animals exhibited a 31% increase in locomotor activity during the dark phase when the most significant effects on RER were observed and could represent an increase in foraging behavior in these mice.

The limitations of indirect calorimetry render it impossible to determine the contributions of specific tissues to the observed increase in fat oxidation. However, certain evidence lends speculation to the liver, skeletal muscle and BAT as major contributors towards the lean phenotype. Metabolic processes in the liver require a considerable amount of energy near equal to that of the brain, and consume approximately 20% of total resting energy expenditure (Klein & Jeejeebhoy, 2002). It is estimated that ~90% of this energy is generated through fatty acid and amino acid oxidation (Müller, 1995). Fatty acid transport within the mitochondrial matrix is regulated by a carnitine‐dependent enzyme shuttle, consisting of carnitine translocase 1 (CPT1) and CPT2 (Fabbrini et al., 2010). While expression levels of the CPT1 isoform *Cpt1a* remained unchanged between groups in liver (Figure 6d), we did observe a significant increase in expression of *Fabp3* in the skeletal muscle as well as an increase of *Cpt1b* that approached significance in this tissue (*p* = 0.06; Figure 5e). *Fabp3* exerts important roles on fatty acid metabolism in the heart and skeletal muscle (Binas et al., 1999; Murphy et al., 2004; Schaap et al., 1999), and has been shown to regulate fatty acid oxidation and thermogenesis of BAT (Vergnes et al., 2011). While increased expression of *Fabp3* alone is not indicative of increased fatty acid binding, uptake and oxidation may be enhanced in skeletal muscle and other tissues to contribute to the lean phenotype. Thus, MPZL3 loss may drive alternate fatty acid distribution and utilization in tissues.

Preliminary studies from our laboratory showed that human MPZL3 co‐localized to APOA1‐containing vesicles in the cytoplasm of HepG2 liver adenocarcinoma cells (Krantz et al., 2015). However, a recent study demonstrated that 55% of MPZL3 localized to the mitochondria and 21% to the cytoplasm in epidermal cells (Bhaduri et al., 2015). An additional proximity analysis demonstrated that MPZL3 primarily interacts with mitochondrial proteins and confirmed MPZL3 mitochondrial localization (Bhaduri et al., 2015). Among proteins identified as interacting with MPZL3 was FDXR, a mitochondrial enzyme that catalyzes the reduction of ferredoxin and is involved in steroid hormone biosynthesis. We observed increased expression of *Fdxr* in WAT and liver and a non‐significant increase in skeletal muscle tissue from *Mpzl3* ASO animals (Figures 5 and 6), which may represent a compensation for the loss of the protein interaction between MPZL3 and FDXR. Intrahepatocellular β‐oxidation can also take place within peroxisomes and microsomes, where it is largely regulated by PPARα (Fabbrini et al., 2010); however, as *Pparα* levels remained unchanged in hepatocytes between groups (Figure 6d), any increased β‐oxidation may likely result from direct effects of *Mpzl3* knockdown on mitochondria. Further studies are necessary to investigate the role of *Mpzl3* in mitochondrial function and potential associated transport of long‐chain fatty acids for β‐oxidation. We also observed downregulation of numerous lipogenic mediators in WAT which is consistent with the specific reductions in fat mass **(**Figure 5 **)**. Lastly, we observed a significant increase in *Dio2* expression in BAT after *Mpzl3* ASO treatment indicating that BAT may be hyperactivated in these mice (Figure 5d). *Dio2* encodes for type II deiodinase which converts inactive thyroid hormone T4 to its active form T3 which stimulates adaptive thermogenesis. Cold exposure significantly increases *Dio2* expression (Yau & Yen, 2020). Interestingly, we observed a reduction in body temperature (Figure 1k) and one could speculate that an increase in BAT thermogenesis could be a compensatory mechanism to preserve body temperature and significantly contribute to an increase in fat oxidation in these mice. Thus, future studies utilizing conditional *Mpzl3* KO mice are necessary to determine the contributions of liver, adipose, skeletal muscle and BAT in *Mpzl3* effects on β‐oxidation and mitochondrial function.

We observed significantly reduced serum cholesterol and triglyceride levels in mice treated with *Mpzl3* ASO (Figures 1 and 2). This was coupled with increased expression of sterol biosynthesis regulators in the liver and WAT (Figures 5c and 6d), including *Hmgcr* and *Srd5a1*; the latter converts testosterone to the more potent androgen dihydrotestosterone (DHT​) (Deslypere et al., 1992), which has been shown to regulate bile acid production (Moverare‐Skrtic et al., 2006). Bile acid synthesis can occur via two pathways, classical or alternative, which are induced by cholesterol 7α‐hydroxylase (*Cyp7a1*) or sterol 27‐hydroxylase (*Cyp27a1*), respectively (Chiang, 2013). In mice fed HED, bile acid synthesis is stimulated through activation of the oxysterol‐activated nuclear receptor LXRα, which in turn induces transcription of *Cyp7a1* or *Cyp27a1* (Chiang, 2013). Bile acids facilitate dietary lipid absorption and cholesterol catabolism (Watanabe et al., 2011), which could provide a mechanism for lipid excretion. We measured expression of two regulators of bile acid synthesis and extrahepatic cholesterol catabolism to bile acids, *Fxr* (*Nr1h4*) and *Cyp7b1* (Figure 6d). Expression of these genes remained unchanged between groups, but further examination of this pathway and an analysis of fecal lipid content is necessary to fully elucidate the role of bile acids as a potential mechanism for excess cholesterol excretion. Indeed, we observed a significant upregulation of the bile acid receptor *Gpbar1* in *Mpzl3* ASO treated mice suggesting that there may be an upregulation of bile acids in *Mpzl3* ASO mice that is driving this upregulation of receptor. However, direct measurements of hepatic bile acid composition still need to be performed. Interestingly, inhibition of SRD5A1 in humans increases liver fat and reduces mobilization of lipids in adipose tissue (Hazlehurst et al., 2016) and raises the possibility that the observed changes may be independent of primary effects on bile acid metabolism. Furthermore, similar to global KO mice, we observed significantly decreased mitochondrial *Ucp2* mRNA expression levels in liver tissue from *Mpzl3* ASO animals (Figure 6). Normal hepatocytes do not endogenously express *Ucp2*, which is found primarily in Kupffer cells where it is believed to alleviate hepatic steatosis through reduction of hepatocellular fat accumulation Baffy, 2005). Consistent with fatty acid accumulation due to decreased *Ucp2* expression, we observed increased Kupffer cell hyperplasia and liver weight in *Mpzl3* ASO animals (Figure 6). Further studies are warranted to investigate the effects of increased Kupffer cell accumulation and underlying consequences, such as inflammation, as a result of *Mpzl3* ablation in the liver. Our initial analysis did not indicate that the increased Kupffer cell number was accompanied by an increase in pro‐inflammatory cytokine expression in this tissue (Figure 6d). Although a more detailed analysis of lipid species in liver of ASO treated mice needs to be performed, it is possible that the increase in Kupffer cell accumulation may be indicative of an elevation in cholesterol synthesis driven by an increase in *Hmgcr*. Moreover, an increase in lipids in this tissue could drive the increase in macrophage accumulation. Although lipid levels were not directly measured in liver, these data are consistent with a local increase in hepatic lipids and the observed reduction in circulating lipids in *Mpzl3* ASO treated mice. Our unpublished findings in global *Mpzl3* KO mice have demonstrated that loss of *Mpzl3* leads to a reduction in serum lipids during a vLDL secretion assay causing us to speculate that alterations in hepatic lipid flux could be driving the observed compensatory changes in lipid metabolism including the local upregulation of cholesterol (T. Czyzyk, B. Worley and T. Auen, unpublished data).

Together, these data demonstrate that *Mpzl3* inhibition is a potential therapeutic approach for treatment of obesity and related comorbid conditions, including insulin resistance, hyperlipidemia and cardiovascular dysfunction. Many questions remain unanswered regarding the protein biology of MPZL3, including post‐translational modification and its molecular function in regulating lipid metabolism. Interactions of MPZL3 with other cellular proteins and signaling pathways have not been fully elucidated in mammalian systems (Bushell et al., 2008; Ramani et al., 2012). A recent proximity analysis demonstrated that MPZL3 interacts with mitochondrial proteins and confirmed both MPZL3 mitochondrial and membrane‐bound localization (Bhaduri et al., 2015). Furthermore, membrane‐localized Ig‐like V‐type‐containing molecules are known mediators of immune cell recruitment during inflammatory processes (Souza & Kane, 2006) such as obesity (Eguchi et al., 2005; Straczkowski et al., 2002), and their expression has been demonstrated to increase following consumption of a fatty meal in humans (Nappo et al., 2002). This suggests that MPZL3 might play a role in the inflammatory response to dietary fat (Cani et al., 2007). In this context it is intriguing and consistent that we observed an increase in macrophages in both WAT and liver in response to *Mpzl3* knockdown. Additional studies are necessary to determine whether *Mpzl3* directly modulates inflammatory pathways in these tissues and will likely necessitate isolation and analysis of specific immune cell populations in liver.

While the relevance of MPZL3 to human obesity has yet to be reported in the literature, two genetic linkage studies have demonstrated that the MPZL3 chromosomal location (11q23.3) is associated with body mass (Hanson et al., 1998; Lindsay et al., 2001) and energy expenditure (Norman et al., 1998) in Pima Indians, an indigenous population displaying extremely high incidences of obesity and type 2 diabetes mellitus (Knowler et al., 1978, 1991). Further studies are warranted to determine how mutations in MPZL3 can modify metabolism in obese, diabetic, or dyslipidemic human populations. While MPZL3 remains a protein of relatively unknown function, determining its mechanism in energy homeostasis will facilitate development of pharmacological approaches to target obesity and related comorbid conditions.

## AUTHOR CONTRIBUTION

Beth L. Worley, Amy C. Arnold., Traci A. Czyzyk conception and design of research; Beth L. Worley, Thomas Auen, Amy C. Arnold., Traci A. Czyzyk performed experiments; Beth L. Worley, Thomas Auen, Traci A. Czyzyk analyzed data; Beth L. Worley, Amy C. Arnold., Nadine Hempel, Traci A. Czyzyk interpreted results of experiments; Beth L. Worley prepared figures; Beth L. Worley drafted manuscript; Beth L. Worley, Amy C. Arnold., Brett P. Monia, Nadine Hempel, Traci A. Czyzyk edited and revised manuscript; Beth L. Worley, Thomas Auen, Amy C. Arnold., Brett P. Monia, Nadine Hempel, Traci A. Czyzyk approved final version of manuscript.

## Data Availability

Data associated with this study are available from the corresponding author upon reasonable request.
